# Essays and Discoveries on the Physiology of the Spinal Marrow

**Published:** 1841-10

**Authors:** 


Art. IV.—*Traités et Découvertes sur la Physiologie de la Moelle Epinière.* Par J. Van Deen, m.d. &c.—*Leide*, 1841. 8vo, pp. 224.
*Essays and Discoveries on the Physiology of the Spinal Marrow.* By J. Van Deen, m.d., &c.—*Leyden*, 1841.

This volume contains a collection of papers, which have been published by the author at different times, from 1838 to the present year, embodying the results of a large number of experiments on the spinal cord and its nerves. The opinions which he deduces from these do not altogether coincide with those usually entertained in this country. For example, he states his present accordance with the proposition of Magendie, that the anterior columns are as much concerned in sensation as in motion; but his earlier views were by no means the same; and the facility with which he changes his interpretation of the same experiments, rather diminishes our confidence in the value of his inferences. The white portion of the anterior column he considers to be the part concerned in movement; and he thinks that he has proved that by the gray matter sensory impressions may be either conveyed to the brain or reflected through the motor nerves. We are left so much in the dark, however, as to the mode in which the persistence of sensibility was determined, that we cannot regard the proof of this and other similar propositions as by any means sufficient. The movements, which the author relies upon as indications of pain, are evidently nothing more than reflex actions; an interpretation which is not forbidden by their adaptive character. In his general conclusion, however, that in the gray matter of the nervous system resides its whole active power, and that the white matter has only the functions of establishing a communication between the central and peripheral organs, we think him much more justified. He fully admits, also, the class of reflex actions as distinguished by Dr. M. Hall; but he refuses his assent to the doctrine of the "distinct system" of excito-motor nerves; maintaining that there is but one kind of centripetal and one of centrifugal fibres. We think it right to give his own summary of his latest conclusions, for the information of such of our readers as may feel an interest in them.

1. That the white substance of the anterior columns ministers solely to motion.

2. That the anterior columns with their gray portion minister to sensation as well as to motion.

3. That the white substance of the posterior columns is concerned solely in sensation.

4. That the posterior columns with their gray substance are also destined solely to sensation.

5. That the white substance of the posterior columns does not require to be continuous with the brain, to communicate to it the impressions received by the posterior roots.

6. That the white substance alone of the posterior columns cannot readily transmit sensory impressions to the brain.

7. That this transmission may readily take place, if the gray substance is still in contact with the white substance of the posterior cords.

8. That the white substance of the anterior columns, without the gray substance, cannot directly communicate voluntary power to the muscles through the posterior roots; but can only excite vibrations in the muscles.

9. That the same conditions which are necessary to cause a real sensation are also necessary for a reflex action; that is to say, as real sensation depends on the posterior roots and columns, and on the gray substance, the impression necessary for reflex action depends on the same parts.

10. That the same conditions required to communicate to the brain a real sensation by the anterior columns are also necessary to transmit by the same columns the movement of reflexion in the direction of the brain; neither one action or the other can take place without the gray matter.

11. That by the gray matter, impressions are communicated from the posterior to the anterior columns.

12. That by the gray matter impressions are communicated from one centripetal fibre to another.

13. That by this medium, the same takes place among the centrifugal fibres; since only a few of the white fibres of the anterior columns seem concerned in transmitting, by the medium of the gray substance, the voluntary impressions proceeding from the brain to the anterior roots.

14. That the centripetal and centrifugal fibres are to be regarded as conductors; and the gray substance as the active centre of the nervous system.

